# Severe metabolic or mixed acidemia on intensive care unit admission: incidence, prognosis and administration of buffer therapy. a prospective, multiple-center study

**DOI:** 10.1186/cc10487

**Published:** 2011-10-13

**Authors:** Boris Jung, Thomas Rimmele, Charlotte Le Goff, Gérald Chanques, Philippe Corne, Olivier Jonquet, Laurent Muller, Jean-Yves Lefrant, Christophe Guervilly, Laurent Papazian, Bernard Allaouchiche, Samir Jaber

**Affiliations:** 1 Intensive Care Unit, Department of Anaesthesia and Critical Care, Saint Eloi Teaching Hospital, Université Montpellier 1, 80 avenue Augustin Fliche, F-34295 Montpellier, Cedex 5, France; 2 Department of Anesthesiology and Critical Care Medicine, Edouard-Herriot Teaching Hospital, hospices civils de Lyon, pavillon G, place d'Arsonval, F-69437 Lyon Cedex 03, France; 3 Medical Intensive Care Unit, Gui-de-Chauliac Teaching Hospital, Université Montpellier 1, 80 avenue Augustin Fliche, F-34295 Montpellier Cedex 5, France; 4 Anesthesiology, Pain Medicine, Emergency and Critical Care Medicine Division, Caremeau Teaching Hospital, Centre Hospitalier Universitaire Nîmes, Place du Professeur Robert Debré, F-30029 Nîmes Cedex 9, France; 5 Medical Intensive Care Unit, Assistance Publique Hôpitaux de Marseille, URMITE CNRS-UMR 6236, Université de la Méditerranée Aix-Marseille II, Chemin des Bourrely, 13915 Marseille cedex 20, France

## Abstract

**Introduction:**

In this study, we sought describe the incidence and outcomes of severe metabolic or mixed acidemia in critically ill patients as well as the use of sodium bicarbonate therapy to treat these illnesses.

**Methods:**

We conducted a prospective, observational, multiple-center study. Consecutive patients who presented with severe acidemia, defined herein as plasma pH below 7.20, were screened. The incidence, sodium bicarbonate prescription and outcomes of either metabolic or mixed severe acidemia were analyzed.

**Results:**

Among 2, 550 critically ill patients, 200 (8%) presented with severe acidemia, and 155 (6% of the total admissions) met the inclusion criteria. Almost all patients needed mechanical ventilation and vasopressors during their ICU stay, and 20% of them required renal replacement therapy within the first 24 hours of their ICU stay. Severe metabolic or mixed acidemia was associated with a mortality rate of 57% in the ICU. Delay of acidemia recovery as opposed to initial pH value was associated with increased mortality in the ICU. The type of acidemia did not influence the decision to administer sodium bicarbonate.

**Conclusions:**

The incidence of severe metabolic or mixed acidemia in critically ill patients was 6% in the present study, and it was associated with a 57% mortality rate in the ICU. In contradistinction with the initial acid-base parameters, the rapidity of acidemia recovery was an independent risk factor for mortality. Sodium bicarbonate prescription was very heterogeneous between ICUs. Further studies assessing specific treatments may be of interest in this population.

## Introduction

"Acidemia" can be defined as the accumulation of protons in the plasma which results in a lower blood pH if secondary responses are overwhelmed. In critically ill patients, acidosis is often the result of a combination of single disorders occurring simultaneously that are commonly known collectively as "mixed acid-base disorders" [1-6].

Although "severe acidemia" is not a universally accepted term, it usually indicates that plasma pH is lower than 7.20 [3,7,8]. Severe acidemia can be critical, especially when an extremely low pH develops quickly. Clinical manifestations of severe acidemia include cerebral edema, seizures, diaphragm dysfunction [9], decreased myocardial contractility, pulmonary vasoconstriction and systemic vasodilatation [3,10,11]. Acidemia is a potentially life-threatening condition, and previous studies have described the incidence and mechanisms of acidosis occurring in the ICU [1,12,13]. Surprisingly, however, these studies failed to focus specifically on severe acidemia. Furthermore, despite the fact that severe acidemia reflects a serious underlying disease that should be treated as soon as possible, the treatment of acidemia by itself with the administration of intravenous buffers remains controversial [7,8,14,15]. Indeed, notwithstanding experts' opinions arguing against treatment with intravenous buffers except in dedicated situations (for example, massive digestive fluid loss during diarrhea or tubular acidosis) [16], 86% of polled US nephrologists indicated that they would prescribe buffers to treat lactic acidosis in patients with a targeted pH above 7.20 [8]. Moreover, the Surviving Sepsis Campaign suggests not treating lactic acidosis when the plasma pH level is above 7.15, but does not give any recommendations for cases where the plasma pH level is below this threshold [7]. To date there have been only two small, prospective, randomized crossover studies assessing the impact of sodium bicarbonate treatment for lactic acidosis on hemodynamic, acid-base and electrolyte changes [14,15]. Both studies included 24 patients and could not address any difference in terms of morbidity and mortality with regard to whether the patients had first been treated with sodium bicarbonate.

Therefore, we had two aims in our study. First, we sought to describe the incidence of severely metabolic or mixed acidemia patients admitted to the ICU. Second, we wanted to describe the outcomes of patients who had been treated at the onset of acidemia (within the first 24 hours of ICU admission) with intravenous sodium bicarbonate compared with those who had not. We hypothesized that severe acidosis within the 24 first hours after ICU admission is an infrequent phenomenon but that its correction rather than its initial severity is associated with prognosis.

## Materials and methods

This prospective, multiple-center study was conducted during a thirteen-month period in five ICUs. In accordance with French law, informed consent was not mandatory, given that this observational study did not modify diagnostic or therapeutic strategies. This study was approved by the Montpellier University Hospital Institutional Review Board and followed the Strengthening the Reporting of Observational Studies in Epidemiology recommendations for reporting observational studies [17].

### Definitions

"Severe acidemia" was defined as a pH below 7.20. Respiratory acidosis, metabolic acidosis and other mixed disorders were categorized using the classical method (namely, the Henderson-Hasselbalch equation) with base excess and corrected anion gap (AG) (corrected AG = (measured AG + (40 g/dl - (albuminemia ÷ 10 g/dl))) [3,18,19].

Therefore, we defined the following specific acidemia as follows. (1) "Severe metabolic acidemia" comprised pH less than 7.20, bicarbonatemia less than 22 mmol/L, base excess less than or equal to 5 mmol/L and expected partial pressure of carbon dioxide (PaCO_2_) = bicarbonatemia × 1.5 + 8 ± 2 mmHg [18]. (2) "Severe respiratory acidemia" was defined as pH less than 7.20, PaCO_2 _greater than 45 mmHg and expected bicarbonatemia = (PaCO_2 _- 40) ÷ 10 + 24). (3) "Severe mixed acidemia" was the classification for secondary response to the primary process outside the expected range.

### Patients

All consecutive patients who experienced severe acidemia within the first 24 hours of their ICU admission were screened, and those who presented with either single metabolic acidemia or mixed metabolic and respiratory acidemia were included. Patients admitted for diabetic ketoacidosis were excluded from the outcome analysis, as their mortality risk in the ICU is very low and may not represent the risk level among the whole population [20]. Furthermore, because one of the aims of the present study was to describe the use of sodium bicarbonate to treat severe acidemia, patients admitted with a single severe respiratory acidemia were not analyzed in detail, because they were considered not to be candidates for that treatment. No guidelines, advice or documentation were given to the intensivists regarding acidemia, treatment or routine daily care.

### Measured parameters

Upon their admission to the ICU, we documented patients' baseline characteristics, SAPS II [21], SOFA [22] severity scores and the reason for admission to the ICU (Table 1). Within the first 48 hours of their ICU stay, we recorded patients' arterial blood pH, PaCO_2_, bicarbonate concentration, standard base excess, creatinine, sodium, potassium, chloride, albumin and lactate (Lac). Apparent strong ion differences were calculated as plasma concentrations: [Na^+^] + [K^+^] + [Mg^2+^] + [Ca^2+^] - [Cl^-^] - [Lac^-^]. Effective strong ion differences were calculated as plasma concentrations: 2.46 × 10^pH 8 ^× PaCO_2 _+ [albumin] × (0.123 × pH - 0.631) + [phosphate] × 0.309 × pH - 0.469] (all concentrations are expressed as mEq/L). The strong ion gap was calculated as the difference between apparent and effective strong ion data [5]. Moreover, we recorded the amount of sodium bicarbonate administered, the amount of required vasopressors and the need for renal replacement therapy, intubation and mechanical ventilation. We also documented the duration of mechanical ventilation and use of vasopressors while in the ICU, the length of ICU stay, and mortality. The primary end point was the mortality rate at ICU discharge. The secondary end points were the amount of time spent on mechanical ventilation in the ICU, the duration of vasopressor use and the overall length of ICU stay.

**Table 1:** Characteristics and main outcomes of the study population

Characteristics	All patients (*n *= 155)	Survivors (*n *= 66)	Nonsurvivors (*n *= 89)	*P *value
Age (years)	63 ± 16	62 ± 16	67 ± 15	< 0.01
Males	99 (64)	41 (62)	58 (65)	0.74
Body mass index (kg/m^2^)	26 ± 6	26 ± 5	27 ± 7	0.43
SAPS II upon ICU admission	67 ± 22	53 ± 16	76 ± 21	< 0.01
SOFA upon ICU admission	10 ± 4	9 ± 4	12 ± 4	< 0.01
Admitting diagnosis group				
Septic shock	54 (35)	25 (37)	29 (33)	0.53
Hemorrhagic shock	16 (11)	5 (8)	11 (12)	0.48
Cardiogenic shock	11 (7)	3 (5)	8 (9)	0.45
Cardiac arrest	19 (12)	5 (8)	14 (16)	0.20
Multiple trauma	10 (6)	5 (8)	5 (6)	0.74
Acute respiratory failure	19 (12)	7 (10)	12 (13)	0.63
Acute renal failure	4 (3)	4 (6)	0 (0)	0.03
Others	22 (14)	12 (18)	10 (11)	0.25
Vasopressor administration upon ICU admission	130 (83)	49 (74)	81 (91)	0.01
Renal replacement therapy within first 24 hours of ICU stay	31 (20)	12 (18)	19 (21)	0.69
Mechanical ventilation within first 24 hours of ICU stay	135 (88)	54 (82)	81 (91)	0.15
Creatininemia upon ICU admission (μmol/L)	200 ± 134	213 ± 166	188 ± 100	0.81
Albuminemia upon ICU admission (g/L)	23 ± 8	25 ± 9	21 ± 7	0.01
Base excess upon ICU admission (mmol/L)	-13.7 ± 6.1	-12.4 ± 6.1	-14.6 ± 5.9	< 0.01
Anion gap upon ICU admission (mmol/L)	23 ± 10	21 ± 10	25 ± 9	< 0.01
Apparent strong ion difference upon ICU admission (mEq/L)	32.4 ± 6.0	35.4 ± 6.5	34.5 ± 5.8	0.16
Effective strong ion difference upon ICU admission (mEq/L)	22.3 ± 6.0	24.9 ± 6.1	22.4 ± 6.0	0.04
Strong ion gap upon ICU admission (mEq/L)	10.4 ± 6.3	10.8 ± 6.4	12.1 ± 5.3	0.21
Sodium bicarbonate administration within first 24 hours of ICU stay	57 (37)	23 (35)	34 (38)	0.73
Amount of sodium bicarbonate administered within first 24 hours of ICU stay in patients receiving sodium bicarbonate (mmol/kg)	3.9 ± 3.2	3.0 ± 2.0	4.0 ± 2.8	0.31
Length of mechanical ventilation (days)	3 [2 to 11]	4 [3 to 6]	2 [2 to 3]	0.04
Length of vasopressor administration (days)	2 [1 to 5]	3 [2 to 4]	2 [2.0 to 2.1]	0.38
Length of ICU stay (days)	5 [2 to 13]	11 [8 to 17]	2 [2 to 3]	< 0.01

SAPS II = Severity Acute Physiology Score II [22]; SOFA = Sequential Organ Failure Assessment [23]. Categorical data are expressed as raw numbers (%). Continuous data are expressed as means ± SD. All other data are medians [range]. Comparisons were made between survivors and nonsurvivors.

### Statistics

Data are expressed as means ± SD or medians and 95% CI for continuous variables and raw numbers and percentages for categorical variables. Two main comparisons were performed. First, we compared survivors to nonsurvivors. Second, we compared patients treated with sodium bicarbonate to those who were not. Continuous data were compared using a Student's *t*-test or a Mann-Whitney *U *test regarding the normality of the population distribution. A Χ^2 ^test was used for categorical variables. Comparisons of several means were performed using repeated-measures analysis of variance and the Tukey's *post hoc *test. Multivariate analyses were performed using a logistic regression model with forward selection procedures to estimate the odds ratio of death (with the 95% CI) after discretization of the continuous variables according to their median values and also to describe the prescription of sodium bicarbonate. Calibration of the logistic model was assessed using the Hosmer-Lemeshow goodness-of-fit test to evaluate the importance of the discrepancy between observed and expected mortality. Each variable associated with a *P *value below 0.20 in the univariate analysis was entered into the model. All values were two-tailed, and *P *< 0.05 was considered statistically significant. Statistical analysis was performed with MedCalc version 9.4.2.0 statistical software (MedCalc Software bvba, Mariakerke, Belgium).

## Results

**Figure 1:**
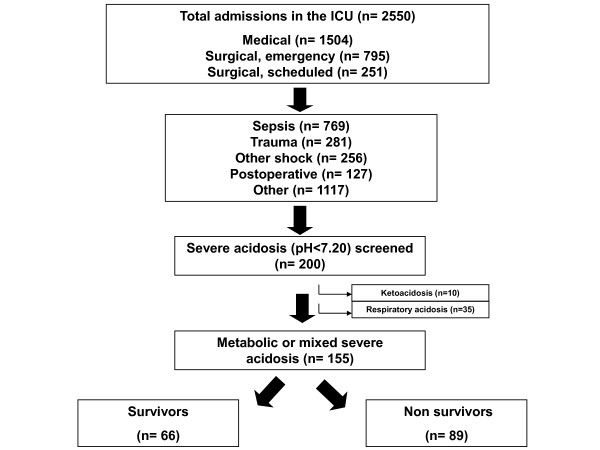
**Study flowchart**.

During the study period, 2, 550 patients were admitted to the five participating ICUs. Among those patients, 200 consecutive patients (8%) with severe acidemia (pH < 7.20) were screened. Among those 200 patients, 35 (18%) exhibited a single respiratory acidemia. Among those 35 patients, 12 died (34%) and they were not further analyzed. Ten patients admitted for ketoacidosis were discharged alive and were not further analyzed (Figure 1).

Next, 155 patients (6% of the 2, 550 screened patients) were eligible for the prognostic analysis. Their characteristics are presented in Table 1. Eighty-four patients (54%) were intubated upon ICU admission, and one hundred thirty-five (88%) were intubated within the first 24 hours of their stay. During their stay, 90% of all patients required mechanical ventilation and vasopressors, 31 (20%) required renal replacement therapy within the first 24 hours of admission and 89 (57%) died during their ICU stay. No difference in terms of diagnosis was observed between survivors and nonsurvivors (Table 1). Conversely, older age, higher SAPS II or SOFA score and vasopressor requirement within the first 24 hours of admission were associated with mortality (Table 1). Although admission pH and bicarbonatemia were similar regardless of intubation status, lactatemia was significantly lower in the nonintubated patients than in those who were intubated (4.1 ± 4.1 mmol/L vs 8.7 ± 6.1 mmol/L; *P *= 0.01).

**Figure 2:**
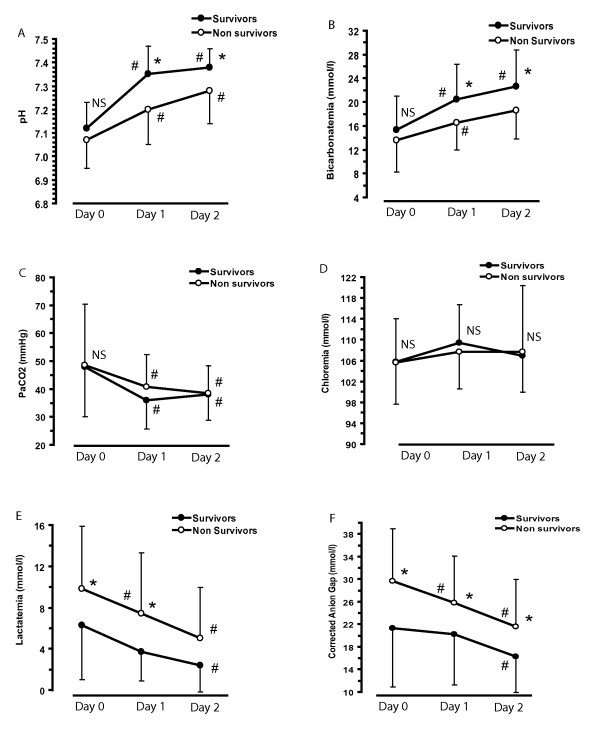
**General features of acid-base metabolism evaluated during the first three days**. **(A) **pH. (B) Bicarbonatemia. **(C) **PaCO_2_. **(D) **Chloremia. **(E) **Lactatemia. **(F) **Corrected anion gap. *P < 0.05, Tukey's *post hoc *analysis between survivors (*n *= 66) and nonsurvivors (*n *= 89). ^#^*P *< 0.05, Tukey's *post hoc *analysis vs day 0. Bars represent standard deviations. NS = not significant. PaCO_2 _= partial pressure of carbon dioxide.

Figures 2A to 2F show the daily evaluation of acid-base and lactate parameters according to ICU survival. On the acid-base parameters, lactatemia and anion gap upon ICU admission were higher in nonsurvivors than in survivors (Table 1 and Figures 2E and 2F). Interestingly, although pH, plasma bicarbonate level and base excess were similar between survivors and nonsurvivors upon ICU admission, plasma pH remained lower in nonsurvivors than in survivors during the first 48 hours of the ICU stay (Additional file 1, Figure S1). We also analyzed acid-base balance according to the use of renal replacement therapy within the first 48 hours after ICU admission. After exclusion of patients receiving sodium bicarbonate infusion, there was not any significant pH variation between day 1 and day 0 according to the prescription of renal replacement therapy. Indeed, pH variation was 0.15 ± 0.14 among the 27 patients on renal replacement therapy compared to 0.18 ± 0.17 among the 73 patients off renal replacement therapy.

Among the 155 included patients, 57 were treated symptomatically with sodium bicarbonate within the first 24 hours of their stay in the ICU (Table 2). No difference was observed between patients treated or not treated with sodium bicarbonate regarding ICU admission diagnosis, acidosis mechanism, severity score and necessity of vasopressors or mechanical ventilation. In logistic regression, base excess was the sole parameter associated with sodium bicarbonate administration (OR = 0.91, 95% CI = 0.85 to 0.97; *P *= 0.005). Among the 98 patients who were not treated with sodium bicarbonate within the 24 first hours of their ICU stay, 6 were finally treated after 24 hours and 3 of them died in the ICU.

**Table 2:** Admission and outcome characteristics of the 155 patients admitted with severe metabolic or mixed acidosis treated with buffers or not at day 0

Characteristics	Sodium bicarbonate administration within first 24 hours of ICU stay (*n *= 57)	No sodium bicarbonate administration within the first 24 hours of ICU stay (*n *= 98)	*P *value
Age (years)	62 ± 16	64 ± 17	0.26
Admitting diagnosis group			
Septic shock	23 (40)	31 (32)	0.30
Hemorrhagic shock	8 (14)	8 (8)	0.28
Cardiogenic shock	2 (4)	9 (9)	0.33
Cardiac arrest	5 (9)	14 (14)	0.45
Others	19 (33)	36 (37)	0.73
SAPS II upon ICU admission	71 ± 23	64 ± 22	0.10
SOFA upon ICU admission	11 ± 4	10 ± 4	0.15
Vasopressor administration upon ICU admission	49 (86)	81 (83)	0.65
Mechanical ventilation upon ICU admission	30 (52)	54 (55)	0.86
Renal replacement therapy within first 24 hours of ICU stay	15 (26)	16 (17)	0.20
Plasma pH upon ICU admission	7.07 ± 0.15	7.10 ± 0.09	0.39
Bicarbonatemia upon ICU admission (mmol/L)	12.6 ± 5.2	15.4 ± 5.6	< 0.01
PaCO_2 _upon ICU admission (mmHg)	41.6 ± 12.6	52.1 ± 22.8	< 0.01
Base excess upon ICU admission (mmol/L)	-15.6 ± 6.3	-12.5 ± 5.7	< 0.01
Anion gap corrected upon ICU admission (mmol/L)	31 ± 11	26 ± 8	0.02
Lactatemia upon ICU admission (mmol/L)	9.3 ± 6.9	7.8 ± 5.5	0.32
Length of vasopressor treatment (days)	3.9 ± 4.7	5.3 ± 8.8	0.35
Length of mechanical ventilation (days)	6.6 ± 10.7	8.3 ± 14.0	0.85
ICU length of stay (days)	10.1 ± 15.5	11.4 ± 15.7	0.32
Mortality in the ICU	34 (60)	55 (56)	0.73

PaCO_2 _= partial pressure of carbon dioxide; SAPS II: Severity Acute Physiology Score II [22]; SOFA = Sequential Organ Failure Assessment [23]. Categorical data are expressed as raw numbers (%). Continuous data are expressed as means ± SD.

Bicarbonate administration ranged from 5% of the patients in one ICU to 55% in another one, depending mostly on the center delivering treatment rather than on the acidemia mechanism. When used, the concentration of sodium bicarbonate was 3.5 ± 3.3 mmol/L within the first 24 hours and ranged from 250 ml of 1.4% solution to 4, 000 ml of 4.2% solution, with no statistical difference observed between survivors and nonsurvivors. The severity of acidemia was not associated with the frequency of sodium bicarbonate prescription, but lower plasma bicarbonate, base excess, PaCO_2 _and higher corrected anion gap were associated with sodium bicarbonate administration (Table 2). The different outcome parameters were not different on the basis of early prescription of sodium bicarbonate (Table 2). Multivariate analysis showed that although plasma pH upon ICU admission was not a predictor of outcome, the persistence of a low plasma pH after 24 hours in the ICU was an independent risk factor for mortality in the ICU (Table 3). Additional details are provided in the Additional file 1.

**Table 3:** Multivariate logistic regression analysis for mortality analysis: results of stepwise selection procedures

Characteristics	Coefficient	SEM	Odds ratio	95% CI	*P *value
SAPS II above 65 upon ICU admission^a^	2.01	0.61	7.53	2.30 to 24.7	< 0.001
Lactatemia upon ICU admission (mmol/L)	0.18	0.08	1.20	1.04 to 1.39	0.01
Plasma pH difference below 0.10 between day 1 and day 0^a^	-1.74	0.59	0.18	0.05 to 0.56	0.003

SAPS II: Severity Acute Physiology Score II [22]. Variables entered into the model were those significantly different between survivors and nonsurvivors in the univariate analysis (Table 1 and Figure 2) upon ICU admission: age above 65 years, SAPS II above 65, SOFA above 10, anion gap, base excess, effective strong ion difference, albuminemia, lactatemia and administration of vasopressors. Plasma level difference between day 1 and day 0 for pH, bicarbonate and base excess and the amount of sodium bicarbonate (in mmol/kg) that were administered within the first 24 hours of ICU stay were also entered into the model. ^a^Discretized according to median value.

## Discussion

The main results of this study can be summarized as follows. First, severe metabolic or mixed acidemia defined by a plasma pH level lower than 7.20 within the first 24 hours of ICU admission was observed in 6% of critically ill patients. Second, severe metabolic or mixed acidemia was associated with an ICU mortality rate of 57%. Third, as opposed to pH value, the rapidity of acidemia correction was associated with mortality. Fourth, sodium bicarbonate prescription within the first 24 hours of acidemia was heterogeneous, depending on the participating ICU, and was not associated with the patient's prognosis.

Several limitations of this study must be identified. First, we defined and classified severe acidemia on the basis of a pH value below 7.20, bicarbonate and base excess [3,18,19], and instead used the physiochemical classification developed by Stewart [1,23]. We chose this strategy because of its widespread use and because it was the easiest way to screen patients [6,24]. Moreover, our study could not demonstrate that acidemia *per se *rather than the underlying disease was the main independent predictive factor in patient outcome. Indeed, our study was an observational, multiple-center study which included all consecutive, heterogeneous patients according to their acid-base status and was not designed to specifically address the impact of sodium bicarbonate on outcome or to address the reasons for sodium bicarbonate prescription. We also could not assess whether very early sodium bicarbonate administration (within the first six hours of acidemia), a different load of buffer (high vs low dose) or administration modalities (slow vs rapid infusion and/or concentrated vs diluted bicarbonate) had any different effects on outcome. However, the present cohort may be used as a tool with which to design a future interventional, randomized study to examine the effects of buffers in severely acidotic critically ill patients.

Although acidosis is a common concern in critically ill patients, studies specifically assessing epidemiology and outcomes of severe acidemia are lacking. Indeed, to our knowledge, the current study is the first prospective, multiple-center investigation focused specifically on severe acidemia occurring in the ICU. Previous studies reported an incidence of acidemia ranging from 14% [18] to 42% [25], but most of the patients in these studies experienced moderate acidemia with a mean base excess of -3. In a large, single-center study of 851 patients for whom arterial lactate levels were evaluated [12], the incidence of metabolic acidosis (defined by a standard base excess below -2 mmol/L) was 64%. In the present study, the incidence of metabolic or mixed severe acidemia was 6% (Figure 1).

The patients included in our study had a mean plasma pH of 7.09 ± 0.11 upon ICU admission. Those patients were defined as critically ill on the basis of their SAPS II and SOFA severity scores and the high rate of vasopressor prescription and mechanical ventilation (Table 1). Renal replacement therapy was initiated in 20% of these patients within the first 24 hours of their ICU stay. More than 50% of the patients died in the ICU (Tables 1 and 2). Upon ICU admission, only lactatemia and corrected anion gap could predict mortality (Figure 2). At day 1, plasma pH, bicarbonate and base excess were lower in nonsurvivors than in survivors (Figure 2). Our results are similar to those reported in a study describing acid-base disorders in 60 septic shock patients, among whom the survivors recovered from metabolic acidemia with correction of unmeasured anions and lactate, whereas nonsurvivors failed to do so [5]. However, the authors in that study screened the patients according to the presence of septic shock rather than the presence and the severity of acid-base disorders and thus could not specifically assess the prognosis of acidotic patients [5]. Another trial included unselected patients and showed that a base excess below -4 mmol/L upon ICU admission was associated with mortality [25]. The association between base excess and mortality was magnified after 24 hours of intensive resuscitation, thus showing the importance of acid-base disorder correction clearance in the critically ill.

In our present study focused on severe metabolic and mixed acidemia, we report that, as opposed to the pH value upon ICU admission, the rapidity of pH recovery was associated with mortality (Table 3). Whether metabolic acidemia is an etiologic contributor to organ dysfunction or just a marker of illness has been debated. Recent findings demonstrate that severe metabolic acidemia is at least a contributory factor to organ dysfunction, favoring cardiac output decrease, arterial dilatation with hypotension, arrhythmia, altered oxygen delivery, respiratory muscle workload increase in spontaneously breathing patients, decreased ATP production and impairment of the immune response [3,16]. Acidemia correction using the administration of a base, primarily in the form of sodium bicarbonate, has naturally become the mainstay of therapy [26,27], but its use continues to generate intense debate [2,28,29]. In our study, we included patients with severe academia, and the prescription of sodium bicarbonate was heterogeneous between the participating ICUs and independent of the mechanism of academia, as shown by chloremia and corrected anion gap values upon ICU admission (Table 1). Lower PaCO_2_, bicarbonatemia and base excess were associated with sodium bicarbonate prescription. Interestingly, our results are similar to those of a recent North American survey which reported that over 60% of intensivists and 80% of nephrologists would consider the use of buffer therapy for the treatment of lactic acidosis [8]. To our knowledge, that survey and our present study are the only investigations that describe the pattern of sodium bicarbonate prescription for severe acidemia in critically ill patients.

When prescribing sodium bicarbonate in the critically ill, the potential side effects of severe acidemia must be balanced with complications related to sodium bicarbonate itself. Indeed, sodium bicarbonate may also be associated with complications such as a transient drop in blood pressure and cardiac output and a decrease in ionized calcium, thus sensitizing the heart to abnormal electrical activity and subsequent arrhythmia [2,3,29]. Moreover, "paradoxical" intracellular acidosis may occur because generated CO_2 _freely diffuses across the cell membrane [2,3,29,30]. In addition, bicarbonate administration can be responsible for hypernatremia, volume overload, the release of proinflammatory cytokines [31] and apoptosis [29]. In the present study, administration of sodium bicarbonate within the first 24 hours of acidemia was not associated with outcome (Tables 2 and 3).

In 2011, there is no human study that has reported any beneficial or detrimental effects of sodium bicarbonate administration when treating patients with severe mixed or metabolic acidemia. Therefore, attention should be first directed toward correcting the underlying basis for the acidemia.

## Conclusions

This study reports for the first time that severe metabolic or mixed acidemia, defined by plasma pH lower than 7.20, occurs within the first 24 hours in the ICU in 6% of critically ill patients. In our study, severe acidemia was associated with a high mortality rate, and, rather than the initial pH value, the rapidity of acidemia correction appeared to be a determinant of outcome. Sodium bicarbonate therapy administration was very heterogeneous between participating ICUs. Further studies are necessary to better assess the role of buffers in this subgroup of critically ill patients.

## Key messages

◆ Severe metabolic and/or mixed acidemia (pH < 7.20) occurred in 6% of patients during the study period in the five participating ICUs.

◆ Severe metabolic and/or mixed acidemia was associated with 57% mortality in the ICU.

◆ Rather than the initial pH value, the rapidity of acidemia correction appeared determine patient outcomes.

◆ Sodium bicarbonate was prescribed for 37% of the patients, was heterogeneous between the participating ICUs and was independent of the mechanism of acidemia.

## Abbreviations

AG: anion gap; SAPS II: Simplified Acute Physiology Score II; SOFA: Sequential Organ Failure Assessment.

## Competing interests

The authors declare that they have no competing interests.

## Authors' contributions

BJ and SJ conceived the study and participated in its design and coordination. TR helped to design the study and to draft the manuscript. CL, PC, GC, CLG and LM collected the data. OJ, JYL, LP and BA helped to correct the manuscript.

## Supplementary Material

Additional file 1 **Figure S1**. Individual values of pH, bicarbonatemia, lactatemia and base excess in survivors and nonsurvivors within the first 24 hours of the ICU stay. Click here for file

## References

[B1] Fencl V Jabor A Kazda A Figge J Diagnosis of metabolic acid-base disturbances in critically ill patients Am J Respir Crit Care Med 2000 162 2246 2251 11112147 10.1164/ajrccm.162.6.9904099

[B2] Kellum JA Acid-base disorders and strong ion gap Contrib Nephrol 2007 156 158 166 17464123 10.1159/000102079

[B3] Kraut JA Madias NE Metabolic acidosis: pathophysiology, diagnosis and management Nat Rev Nephrol 2010 6 274 285 10.1038/nrneph.2010.33 20308999

[B4] Kaplan LJ Cheung NH Maerz L Lui F Schuster K Luckianow G Davis K A physicochemical approach to acid-base balance in critically ill trauma patients minimizes errors and reduces inappropriate plasma volume expansion J Trauma 2009 66 1045 1051 10.1097/TA.0b013e31819a04be 19359913

[B5] Noritomi DT Soriano FG Kellum JA Cappi SB Biselli PJ Liborio AB Park M Metabolic acidosis in patients with severe sepsis and septic shock: a longitudinal quantitative study Crit Care Med 2009 37 2733 2739 10.1097/CCM.0b013e3181a59165 19885998

[B6] Adrogue HJ Gennari FJ Galla JH Madias NE Assessing acid-base disorders Kidney Int 2009 76 1239 1247 10.1038/ki.2009.359 19812535

[B7] Dellinger RP Levy MM Carlet JM Bion J Parker MM Jaeschke R Reinhart K Angus DC Brun-Buisson C Beale R Calandra T Dhainaut JF Gerlach H Harvey M Marini JJ Marshall J Ranieri M Ramsay G Sevransky J Thompson BT Townsend S Vender JS Zimmerman JL Vincent JL Surviving Sepsis Campaign: International guidelines for management of severe sepsis and septic shock: 2008 Intensive Care Med 2008 34 17 60 10.1007/s00134-007-0934-2 18058085 PMC2249616

[B8] Kraut JA Kurtz I Use of base in the treatment of acute severe organic acidosis by nephrologists and critical care physicians: results of an online survey Clin Exp Nephrol 2006 10 111 117 10.1007/s10157-006-0408-9 16791396

[B9] Jaber S Jung B Sebbane M Ramonatxo M Capdevila X Mercier J Eledjam JJ Matecki S Alteration of the piglet diaphragm contractility *in vivo *and its recovery after acute hypercapnia Anesthesiology 2008 108 651 658 10.1097/ALN.0b013e31816725a6 18362597 PMC2789322

[B10] Arieff AI Gertz EW Park R Leach W Lazarowitz VC Lactic acidosis and the cardiovascular system in the dog Clin Sci (Lond) 1983 64 573 580 6839663 10.1042/cs0640573

[B11] Adrogue HJ Madias NE Management of life-threatening acid-base disorders: first of two parts N Engl J Med 1998 338 26 34 10.1056/NEJM199801013380106 9414329

[B12] Gunnerson KJ Saul M He S Kellum JA Lactate versus non-lactate metabolic acidosis: a retrospective outcome evaluation of critically ill patients Crit Care 2006 10 R22 10.1186/cc3987 16507145 PMC1550830

[B13] Tuhay G Pein MC Masevicius FD Kutscherauer DO Dubin A Severe hyperlactatemia with normal base excess: a quantitative analysis using conventional and Stewart approaches Crit Care 2008 12 R66 10.1186/cc6896 18466618 PMC2481449

[B14] Cooper DJ Walley KR Wiggs BR Russell JA Bicarbonate does not improve hemodynamics in critically ill patients who have lactic acidosis: a prospective, controlled clinical study Ann Intern Med 1990 112 492 498 2156475 10.7326/0003-4819-112-7-492

[B15] Mathieu D Neviere R Billard V Fleyfel M Wattel F Effects of bicarbonate therapy on hemodynamics and tissue oxygenation in patients with lactic acidosis: a prospective, controlled clinical study Crit Care Med 1991 19 1352 1356 10.1097/00003246-199111000-00008 1935152

[B16] Kellum JA Disorders of acid-base balance Crit Care Med 2007 35 2630 2636 10.1097/01.CCM.0000286399.21008.64 17893626

[B17] von Elm E Altman DG Egger M Pocock SJ Gotzsche PC Vandenbroucke JP The Strengthening the Reporting of Observational Studies in Epidemiology (STROBE) statement: guidelines for reporting observational studies Lancet 2007 370 1453 1457 10.1016/S0140-6736(07)61602-X 18064739

[B18] Dubin A Menises MM Masevicius FD Moseinco MC Kutscherauer DO Ventrice E Laffaire E Estenssoro E Comparison of three different methods of evaluation of metabolic acid-base disorders Crit Care Med 2007 35 1264 1270 10.1097/01.CCM.0000259536.11943.90 17334252

[B19] Kellum JA Clinical review: reunification of acid-base physiology Crit Care 2005 9 500 507 10.1186/cc3789 16277739 PMC1297616

[B20] Boord JB Graber AL Christman JW Powers AC Practical management of diabetes in critically ill patients Am J Respir Crit Care Med 2001 164 1763 1767 11734423 10.1164/ajrccm.164.10.2103068

[B21] Le Gall JR Lemeshow S Saulnier F A new Simplified Acute Physiology Score (SAPS II) based on a European/North American multicenter study JAMA 1993 270 2957 2963 10.1001/jama.270.24.2957 8254858

[B22] Vincent JL de Mendonca A Cantraine F Moreno R Takala J Suter PM Sprung CL Colardyn F Blecher S Use of the SOFA score to assess the incidence of organ dysfunction/failure in intensive care units: results of a multicenter, prospective study. Working group on "sepsis-related problems" of the European Society of Intensive Care Medicine Crit Care Med 1998 26 1793 1800 10.1097/00003246-199811000-00016 9824069

[B23] Stewart PA Modern quantitative acid-base chemistry Can J Physiol Pharmacol 1983 61 1444 1461 10.1139/y83-207 6423247

[B24] Kurtz I Kraut J Ornekian V Nguyen MK Acid-base analysis: a critique of the Stewart and bicarbonate-centered approaches Am J Physiol Renal Physiol 2008 294 F1009 F1031 10.1152/ajprenal.00475.2007 18184741

[B25] Smith I Kumar P Molloy S Rhodes A Newman PJ Grounds RM Bennett ED Base excess and lactate as prognostic indicators for patients admitted to intensive care Intensive Care Med 2001 27 74 83 10.1007/s001340051352 11280677

[B26] Narins RG Cohen JJ Bicarbonate therapy for organic acidosis: the case for its continued use Ann Intern Med 1987 106 615 618 3103511 10.7326/0003-4819-106-4-615

[B27] Narins RG Emmett M Simple and mixed acid-base disorders: a practical approach Medicine (Baltimore) 1980 59 161 187 6774200 10.1097/00005792-198005000-00001

[B28] Gunnerson KJ Kellum JA Acid-base and electrolyte analysis in critically ill patients: are we ready for the new millennium? Curr Opin Crit Care 2003 9 468 473 10.1097/00075198-200312000-00002 14639065

[B29] Sabatini S Kurtzman NA Bicarbonate therapy in severe metabolic acidosis J Am Soc Nephrol 2009 20 692 695 10.1681/ASN.2007121329 18322160

[B30] Boyd JH Walley KR Is there a role for sodium bicarbonate in treating lactic acidosis from shock? Curr Opin Crit Care 2008 14 379 383 10.1097/MCC.0b013e3283069d5c 18614899

[B31] Nichol AD O'Cronin DF Howell K Naughton F O'Brien S Boylan J O'Connor C O'Toole D Laffey JG McLoughlin P Infection-induced lung injury is worsened after renal buffering of hypercapnic acidosis Crit Care Med 2009 37 2953 2961 10.1097/CCM.0b013e3181b028ce 19773647

