# Supplementary figures and images for: Severe metabolic or mixed acidemia on intensive care unit admission: incidence, prognosis and administration of buffer therapy. a prospective, multiple-center study
Source: Crit Care. 2011 Oct 13;15(5):R238. doi: 10.1186/cc10487 (PMC3334789; doi:10.1186/cc10487)

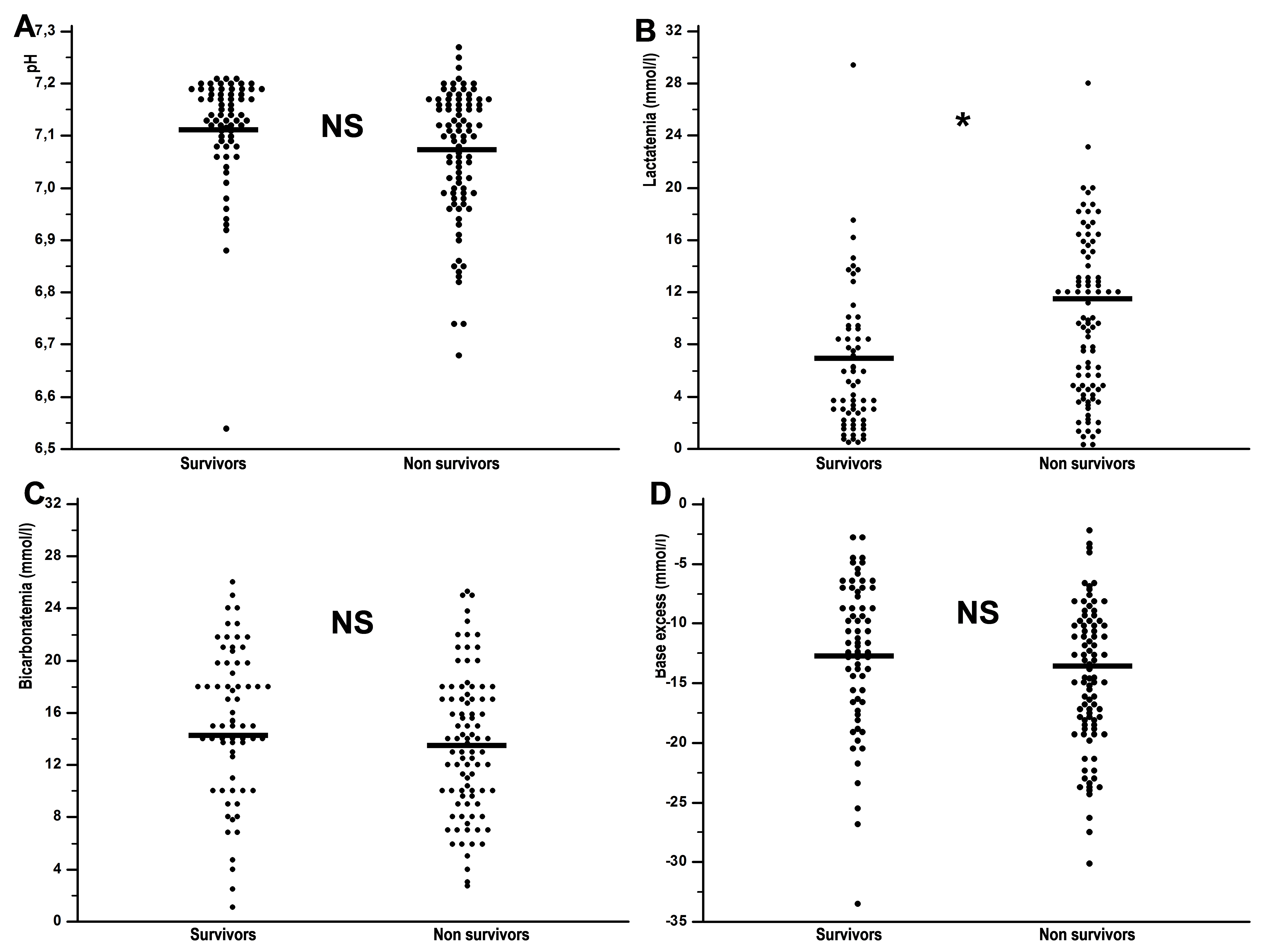

Supplement: Additional file 1 — Figure S1. Individual values of pH, bicarbonatemia, lactatemia and base excess in survivors and nonsurvivors within the first 24 hours of the ICU stay. [file cc10487-S1.TIFF]
